# Identification of therapeutic targets in osteoarthritis by combining heterogeneous transcriptional datasets, drug-induced expression profiles, and known drug-target interactions

**DOI:** 10.1186/s12967-024-05006-z

**Published:** 2024-03-15

**Authors:** Maria Claudia Costa, Claudia Angelini, Monica Franzese, Concetta Iside, Marco Salvatore, Luigi Laezza, Francesco Napolitano, Michele Ceccarelli

**Affiliations:** 1 Biogem s.c.ar.l, Ariano Irpino, Italy; 2 Dipartimento di Ingegneria Elettrica e delle Tecnologie dell’Informazione, Università di Napoli Federico II, Napoli, Italy; 3 Istituto per le Applicazioni del Calcolo, Consiglio Nazionale delle Ricerche, Napoli, Italy; 4 IRCCS SYNLAB SDN, Napoli, Italy; 5 Department of Public Health Sciences, Miller School of Medicine, University of Miami, Miami, FL USA; 6 Dipartimento di Scienze e Tecnologie, Università degli Studi del Sannio, Benevento, Italy

**Keywords:** OA, Cartilage, Consensus signature, Network, Risk score, Drug prediction

## Abstract

**Background:**

Osteoarthritis (OA) is a multifactorial, hypertrophic, and degenerative condition involving the whole joint and affecting a high percentage of middle-aged people. It is due to a combination of factors, although the pivotal mechanisms underlying the disease are still obscure. Moreover, current treatments are still poorly effective, and patients experience a painful and degenerative disease course.

**Methods:**

We used an integrative approach that led us to extract a *consensus* signature from a meta-analysis of three different OA cohorts. We performed a network-based drug prioritization to detect the most relevant drugs targeting these genes and validated in vitro the most promising candidates. We also proposed a risk score based on a minimal set of genes to predict the OA clinical stage from RNA-Seq data.

**Results:**

We derived a *consensus* signature of 44 genes that we validated on an independent dataset. Using network analysis, we identified Resveratrol, Tenoxicam, Benzbromarone, Pirinixic Acid, and Mesalazine as putative drugs of interest for therapeutics in OA for anti-inflammatory properties. We also derived a list of seven gene-targets validated with functional RT-qPCR assays, confirming the in silico predictions. Finally, we identified a predictive subset of genes composed of *DNER, TNFSF11, THBS3, LOXL3, TSPAN2, DYSF, ASPN* and *HTRA1* to compute the patient’s risk score. We validated this risk score on an independent dataset with a high AUC (0.875) and compared it with the same approach computed using the entire *consensus* signature (AUC 0.922).

**Conclusions:**

The *consensus* signature highlights crucial mechanisms for disease progression. Moreover, these genes were associated with several candidate drugs that could represent potential innovative therapeutics. Furthermore, the patient’s risk scores can be used in clinical settings.

## Background

Osteoarthritis (OA) is the predominant joint disease in middle-aged and older people, and the risk of being affected dramatically increases with aging [1–4]. Often labeled “wear and tear” disorder, it is defined as a hypertrophic, complex, and multifactorial condition affecting the whole joint (cartilage, subchondral bone, synovial membrane and fluid, ligaments, adjacent joint muscles), characterized by stiffness, resulting in reduced function, pain on movements and crepitus [2, 4]: the most affected sites are knees, hips, hands, and spine likewise multiple joints are commonly involved [4]. Overall, recent studies show that 18% of women and 9.6% of men over 60 have symptomatic OA [2], where most people over 75 show radiographic evidence of the disease [3]. Obesity, female gender, prior joint trauma, performing specific activities, and acquired or congenital anatomic abnormalities represent additional factors of risk. However, the causes of the onset of the disease are complex and multifactorial, encompassing biochemical, environmental, genetic, and systemic factors [2]. Evidence of a genetic influence comes from several sources, including epidemiological studies of family history and exploration of rare genetic disorders related to OA, like chondrodysplasias: alterations in genes such as *VDR, COL2A, AGC1, IGF-1* are supposed to be involved in the hereditability component of the disorder, which is overall estimated to be greater than $50\%$ [5]. The impact of the environment and lifestyle can also be determinant. Indeed, diet, alcohol consumption, and smoking can also have a role, but additional studies are needed to assess the actual influence of these factors on the OA outbreak [2]. People affected have a painful and degenerative course in most cases. Currently, available treatments aim for pain control and improved quality of life, still not being entirely adequate [6]. This scenario forces the patient to experience an extremely disabling condition, both physically and psychologically. Indeed, OA represents a pathology with a high social impact, also burdening the healthcare system [1].

Research in the field of OA focuses on different topics: on the one hand, it is crucial to understand the fundamental mechanisms underlying the onset and evolution of OA. On the other hand, the research on new therapeutics and strategies for effective patient treatments remain urgent. To date, the use of different technologies, encompassing the exploration of various omics, has made it possible to shed some light on the pivotal processes involved. It has been established that inflammatory signaling and extracellular matrix (ECM) remodeling are the predominant activated mechanisms [1].

Numerous studies have demonstrated the presence of a considerable number of Single Nucleotide Polymorphisms (SNPs) associated with the onset of OA pathology, particularly in non-coding regions [7]: this represents evidence of the decisive part played by transcription dysregulation in the establishment and development of the OA phenotype, where modifications of the methylation pattern and chromatin accessibility of these specific loci are determinant [8, 9]. The role of non-coding transcripts is also relevant. Several studies confirmed miRNAs as modulators of cartilage homeostasis, down-regulated in the affected patients. Some of these, like miR-204, miR-211 miR-335-5, and miR-93 activate the autophagic process and inhibit the inflammatory response, interfering with the TNF-$\alpha$ and interleukins mediated pathways [10–13]. Across the biological programs potentially altered in the OA patient, TGF$\beta$ and WNT signaling are crucial: the TGF$\beta$-FOXO1 axis activated by TAK1 and the TGF$\beta$-pSMAD2/3-FBXO6 axis are involved in the development and homeostasis of healthy joint cartilage, regulating autophagy and ubiquitination [14–16] whereas hyperactivation of the WNT program has been associated with chondrocyte hypertrophy and OA [16, 17]. Regarding clinical research, in recent years, scientists have focused on regenerative therapy, where various studies report how the use of stem cells (both deriving from healthy tissue and mesenchyma) can bring the joint in the initial stage of OA to a good level of regeneration [18–21].

Despite the achievements and results, it has not been clarified yet which genes involved in the maintenance and progression of OA are the master regulators of a dysfunctional and degenerative phenotype and how they can be regulated using available drugs [22]. This gap can be due to the variability introduced by the various experimental strategies and the specificities of the different cohorts analyzed. Our study, using a meta-analysis of three available cohorts, identified a “consensus” signature of 44 differentially expressed genes in OA that recapitulate the main OA patient’s transcriptional profile. Based on such a signature, we performed two types of analysis: *disease–centric* and *patient–centric*. In the *disease–centric* approach, we built a Protein–Protein Interaction (PPI) network encompassing both consensus genes and relevant drug targets, revealing interesting drug-genes interactions and suggesting potential novel treatments. Based on the network, we also selected a list of genes for in vitro validation on human chondrocytes. The *patient–centric* strategy aimed to define a *risk score* using the transcriptional data to estimate the severity of the OA disease of each patient. Indeed, for this purpose, we identified a more incisive sub-signature of eight genes to predict the OA status effectively and rapidly, suggesting its potential use in clinical applications. Our results indicate that the selected genes retain the main features of the OA transcriptional profile. Moreover, further studies on the prioritized drugs can also help the clinical research for new therapeutics.

## Materials and methods

**Fig. 1:**
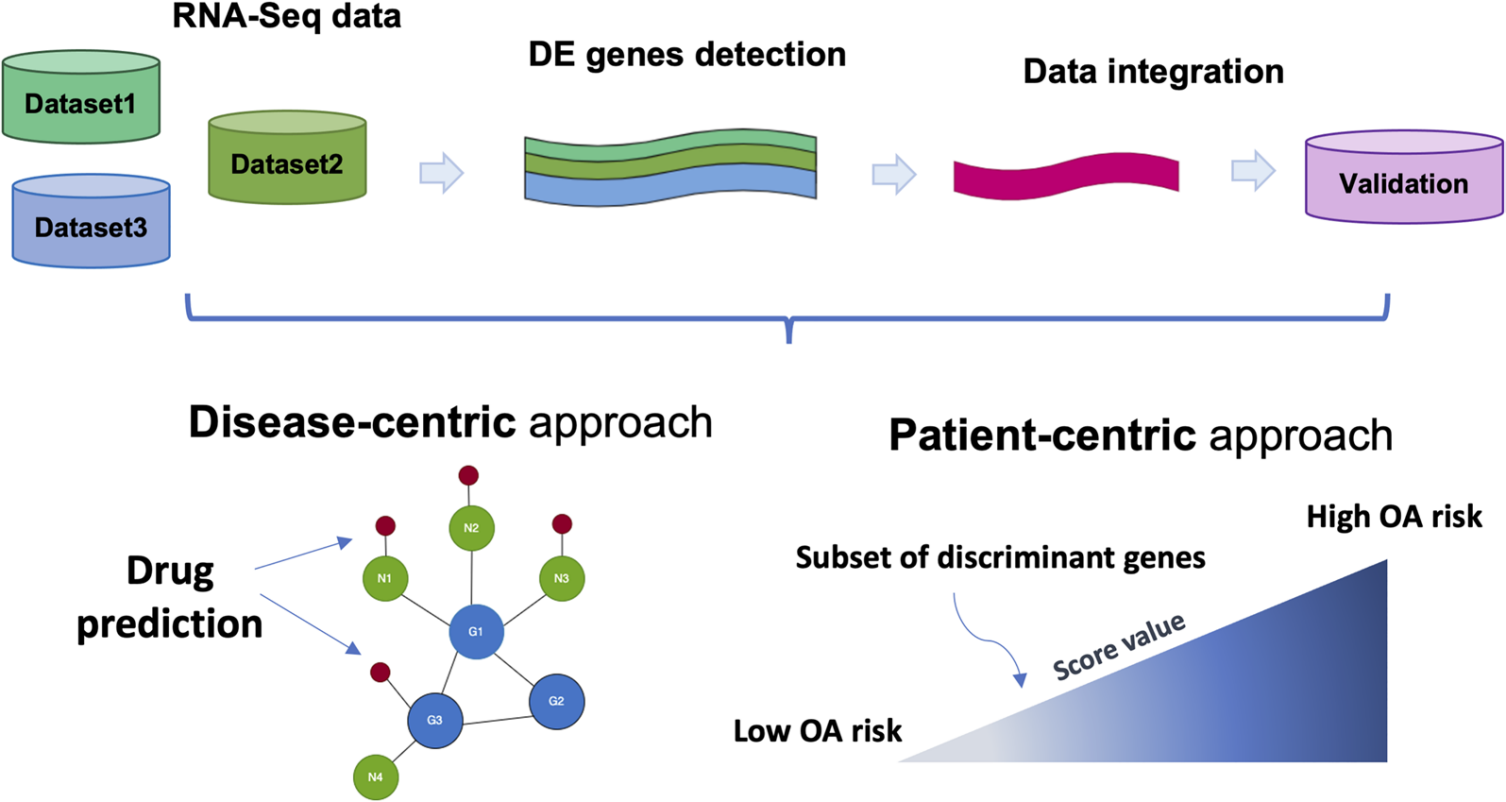
Pipeline workflow. The workflow is summarized in this figure

Figure 1 illustrates the workflow proposed in this work. Starting from a meta-analysis of RNA-seq datasets from three different cohorts, we set up two pipelines for studying the profile of the OA patients from two points of view: a *disease–centric* approach, in which we prioritized potential drug targets among the differentially expressed genes, and a *patient–centric* strategy, aimed to propose a risk score associated with the severity of the disease based on the expression of few relevant genes. We confirmed the biological findings using an independent dataset and experimentally validated the expression of 7 of the 44 genes *in vitro* on human OA chondrocytes.

As a preliminary step, we collected data from the three cohorts in Table 1 (Dataset 1–3), independently pre-processed, and identified differentially expressed genes (DE) associated with OA. In the meta-analysis phase, we integrated the three lists of genes from the DE analysis to return a *consensus* signature of genes able to capture biological information about the OA transcriptomic profile. For reproducibility purposes, we have created a repository accessible at https://github.com/ceccarellilab/MEDIAproject. The repository contains code related to all the pre-processing steps (enriched by further exploratory analyses), the consensus signature extraction, enrichment analyses, and the patient-centric approach workflow. The user will find a dedicated vignette and related useful files for each section to support the analyses. We provided in the repository an R markdown and a Python notebook with the related code to reproduce the disease-centric approach. Further, we added details about the software for the network construction.

**Table 1:** Datasets used in this study

Name	Experiment	Cohort	References
Dataset 1	Single cell-paired	3 OA/N	Chou et al. [23]
Dataset 2	Bulk-paired	12 OA/N	Steinberg et al. [24]
Dataset 3	Bulk-unpaired	60 OA, 10 N	Soul et al. [25]
Dataset 4/Validation	Bulk-unpaired	20 OA, 18 N	Fisch et al. [26]

For each dataset, the experiment design, cohort size, and reference are specified

### Single-cell RNA-Seq cohort (Dataset 1) data collection and pre-processing

We downloaded the raw gene expression count matrix, genes, and barcodes from Gene Expression Omnibus (GEO), id: GSE152805 [23]. This cohort includes data from three patients (one male and two females) suffering from knee OA, with an average age of 67.7 years. These patients had been previously extracted by chance from a cohort of 22 patients subjected to total knee replacement for Medial Compartment OA [23]. For this work, we considered only the transcriptional profiles of 26,228 chondrocytes. We grouped them into two conditions according to the available annotations: (i) derived from damaged tissue (medial region of the tibia, MT, 11,603 cells) or (ii) from minimally damaged tissue (outer lateral region of the tibia, OLT, 14,625 cells). After performing quality control steps using the standard Seurat pipeline, we retained 23,752 cells [27–30]. After that, we normalized and integrated the data to remove the potential batch effects by using the default procedure from the Seurat R package. For this aim, we extracted 8,183 features to use as anchors for the integration by using the SelectIntegrationFeatures and FindIntegrationAnchors functions, and we applied the IntegrateData function. Then, we applied scaling and dimension reduction. We used the pseudo-bulk approach in the R package muscat [31] with the raw RNA assay to identify DE genes between MT and OLT conditions; in particular, we aggregated the data for each patient using the aggregateData function, with “sum” as the summary aggregation parameter. We created a DESeq2 object and used the *patient* identifier to deal with the patient effect and perform a DE analysis [32]. We adjusted the p-value using the BH correction (FDR) [33] and defined DE those genes with $\log _2$ Fold Change (FC) less than − 1.5 or greater than 1.5 and adjusted p-value $\le 0.05$. Detailed information about the computational procedure used to analyze this cohort is available at https://github.com/ceccarellilab/MEDIAproject. The user could find additional explorative analyses here.

### Bulk RNA-Seq data collection and pre-processing

We collected bulk RNA-Seq data from two distinct cohorts. The first consists of a paired cohort (i.e., with healthy and OA samples from the same individual), and the second includes healthy and OA samples from independent individuals. Additional details for each dataset are provided in the following subsections. For more details about the procedure employed to analyze these cohorts, see the vignette (https://github.com/ceccarellilab/MEDIAproject), where one can access other explorative analyses (i.e., PCAs, Volcano plots, enrichment analyses) done on DE genes for both the cohorts.

#### Paired bulk RNA-Seq cohort (Dataset 2)

We downloaded the aligned (reference genome hg19) bam files from the European Genome-phenome Archive (EGA), id: EGAD00001001331 [24]. We quantified the gene expression raw counts using the featureCounts function from the R package Rsubread [34] using as annotation the hg19.ensGene.Chr.gtf.gz file downloaded from the UCSC genome browser.^1^ This cohort profiled paired samples (healthy and damaged cartilage/endochondral bone) from 12 patients (2 females and 10 males) affected by hip and knee OA and underwent total joint replacement. According to the study by Steinberg and colleagues, these patients did not have any malignancies, infections, or knee injuries in the previous 5 years. Additionally, they had not used glucocorticoids in the last 6 months [24]. We filtered out low-expressed genes from the count matrix, retaining only genes with a minimum of 15 counts for at least 50% of the patients. Then, we retrieved the gene symbols using GenomicFeatures [35] and EnsDb.Hsapiens.v75 [36] as an annotation. To deal with gene symbol duplicates, we used the mean counts across the detected isoforms as gene counts for each sample. We accounted for the patient effect, including the factor variable *Donor* in the design matrix, and then we applied the DESeq2 approach to perform a DE analysis. We corrected the p-value for multiple tests [33] and declared DE those genes with $\log _2FC \le -0.8$ or $\log _2FC \ge 0.8$ and FDR $\le 0.05$.

#### Unpaired bulk RNA-Seq cohort (Dataset 3)

We downloaded the raw-counts data related to cartilage tissues from the folder “data” in the GitHub repository^2^ associated with the study of Soul and colleagues [25]. We used the *txi.RData* and *patientDetails_all_withMed.csv* files, i.e., the RNA-Seq data pre-processed with tximport R package [37] and associated patients’ meta-data. This cohort includes 60 Knee OA-affected patients (27 females and 33 males) and 10 non-OA-affected patients (9 males and 1 female) with peripheral vascular disease and no history or clinical sign of OA, joint disease, or joint trauma [25], with an overall average age of 70.7 years (55–86). All patients were subjected to total knee replacement and were diagnosed with predominant Medial compartment OA; as specified by Soul et al., affected samples were isolated from the posterior lateral condyle (PLC), whereas non-OA aliquots were extracted from amputation above the knee [25]. We extracted the integer counts by using the DESeqDataSetFromTximport function, then we filtered out the low-expressed genes, retaining those with a minimum of 5 counts in at least 50% of the samples. We retrieved the gene symbols using EnsDb.Hsapiens.v79 [38] as an annotation. We processed all the duplicated genes’ isoforms as done for Dataset 2. We removed the known batch effect by adding the *Batches* variable to the design matrix and used DESeq2 to apply a DE analysis; we corrected the p-values [33] and selected the DE genes using the same criteria as in Dataset 1, and then we removed 5 genes for which DESeq2 did not provided a p-value. For the patient-centric pipeline, we retrieved transcript per million (TPM) values provided [25]. For more details on all the pre-processing steps on TPMs, see the “Data pre-processing to perform the patient-centric strategy” paragraph.

### Validation cohort data collection and pre-processing (Dataset 4)

As validation dataset, we downloaded the raw-counts as two spreadsheet files directly from GEO, id: GSE114007. This dataset, described by Fisch et al. [26], comprises knee cartilage tissues from 18 healthy samples (5 females and 13 males with an average age of 38, with no history of knee injuries or joint diseases) and 20 OA-affected patients (12 females, 8 males, average age of 66). In particular, normal samples were collected from tissue banks, where they were processed from 24 h to 48 h post-mortem. Affected aliquots were harvested during the knee replacement surgery procedure [26].

For this dataset, we collected the complete list of genes from the DE analysis (OA vs Normal) from the supplementary file NIHMS992829−supplement−Suppl_matl.pdf [26] considering DE those genes with $\log _2FC \le -1.5$ or $\log _2FC \ge 1.5$ and FDR $\le 0.05.$ This list was crucial to validate the consensus signature we obtained (see next section) and also evaluate the robustness of the model implemented in the patient-centric approach (see “Models set-up for risk score computation” section).

Indeed, we used these data to compute a Venn plot between consensus signature and DE genes from this cohort by using the ggvenn function of the homonymous R package [39] and also to perform enrichment analysis (see “Enrichment analyses” section for details). Moreover, we validated the significance of the Venn plot results by the hypergeometric test [40]. For this dataset we retained genes with no less than 5 counts in at least the 50% of the samples, normalized for variance using the vst function from DESeq2 [32] and then batch-corrected these data for the *condition* variable using the removeBatchEffect function from the Limma R package [41].

To assess the validity of the consensus signature, we applied unsupervised clustering on the z-score transformed normalized data of the consensus genes available for this cohort. We plotted the heatmap using the heatmap3 R package [42], maintaining the default clustering parameters (distance calculation method = “euclidian” and hierarchical clustering method = “complete”). To estimate the validity of our risk score in this dataset, we also derived the TPM values using the raw-count data (see “Data pre-processing to perform the patient-centric strategy” section). More detailed information about these procedures and other explorative analyses are available in the vignette (https://github.com/ceccarellilab/MEDIAproject).

### Meta-analysis and identification of a consensus signature

We performed a meta-analysis of the three cohorts (Dataset 1, Dataset 2, Dataset 3) by combining the p-values of the respective gene lists returned from the DE analysis using the Fisher approach [43]. Before combining the p-values, we set to 0 the $\log _2FC$ of all the genes with a slight variation (i.e., $-0.1 \le \log _2FC \le 0.1$). We replaced with 0 the NA $\log _2FCs$ of the genes missing in one of the three cohorts. This was done to compute a weighted combined median $\log _2FC$ that would be smaller in absolute value than the same calculated by just averaging the only two $\log _2FC$ values available. By doing this, we accounted for the absence of a gene in one of the datasets. We flagged those genes with a difference in sign on the $\log _2FC$ across the case studies, and we removed those with no change for all three cohorts. We used Fisher’s combined probability test to combine the (not adjusted) p-values of all genes measured in at least two of the three datasets.

We set the combined p-value to 1 for those genes showing discordant signs of $\log _2FC$ across the cohorts. Then, we assigned a combined $\log _2FC$ value as the median of the available $\log _2FC$ values from the respective DE analyses. Finally, we corrected for multiplicity using the BH approach [33] and obtained adjusted combined p-values. Genes with an adjusted combined p-value (FDR) $\le 0.01$ and a median combined $\log _2FC \le -1.5$ or $\log _2FC \ge 1.5$ are selected as part of the consensus signature (and declared as down and up-regulated genes in OA samples, respectively).

We added a dedicated vignette showing in detail the code used for these analyses (see “Materials and methods” section).

### Enrichment analyses

To understand the molecular mechanisms leading to OA, we used the Gene Set Enrichment Analysis (GSEA) approach [44] to retrieve the GO:BP (Gene Ontology–Biological Processes) terms and REACTOME pathways enriched for each of the three DE lists. To identify the biological programs enriched by the up-regulated consensus genes, we applied an over-representation analysis (ORA) using the Hypergeometric test [40]. We set a value of 0.15 as the FDR cutoff for GSEA and 0.05 for the ORA [33]. We used Enrichplot [45], msigdbr [46], and clusterProfiler [47] to perform these analyses.

We assessed the enrichment of the consensus signature in the Validation cohort by obtaining the Running (Enrichment Score) plot (FDR $\le 0.01$). We first performed a GSEA analysis of the Validation DE genes using the consensus signature as the “gene set”, then we computed the Running plot using the gseaplot2 function by clusterProfiler.

### Disease-centric approach: drug prioritization

To perform drug prioritization, we scored drugs based on their ability to induce an opposite transcriptional response compared to that induced by the disease. This was achieved through a pathway-based approach, which enabled us to abstract the analysis from individual genes to molecular activities while also working around the problem of matching the gene expressions measured by different platforms. In particular, we adapted previously published related approaches that have been used to identify drugs inducing a desired transcriptional effect in a pathway-centric space instead of a gene-centric space. The performance of the pathway-centric approach has been previously assessed in diverse contexts, for example, in the identification of small molecules increasing the efficiency of stem cell differentiation protocols [48] or inducing partial rescue of mutated proteins in a cellular model of cystic fibrosis [49]. In further detail, this approach applies a GSEA-like algorithm to evaluate the enrichment of a large collection of pathways against a given gene expression profile. Once the enrichment scores are computed, they are used in place of the gene expression values to form a pathway-based expression profile (PEP). Techniques similar to those applied to gene-centric profiles can then be applied to the PEPs, including the GSEA, which can be renamed Pathway Set Enrichment Analysis (PSEA) in this context. The PSEA evaluates how much a set of pathways tends to fall towards the top or the bottom of a PEP. In this work, we adapted the PSEA methodology [49] to account for two sets of pathways simultaneously. We selected all the pathways from the GO:MF (Gene Ontology–Molecular Function) category involving any of the genes in the consensus signature. In particular, we performed this selection separately for the up-regulated (“up set”) and down-regulated (“down set”) genes. Next, using the Gep2Pep R package [50], we scored the drug-induced gene expression profiles from the Connectivity Map (CMap), applying PSEA separately against the up and down sets. We then ranked all the CMap drugs according to their PSEA scores so that the top drugs had positive (negative) enrichment values for the pathways involving down- (up-) regulated genes. Finally, we obtained a single score for each drug by computing the average of such ranks.

#### Network analysis

To elucidate the potential mechanisms of action of interest characterizing the top prioritized drugs, we connected the molecular context with the consensus signature. For this purpose, we extracted drug target information from the Therapeutic Target Database [51]. Next, we used the STRING [52] PPI network to find paths connecting each gene in the signature to each known gene target of the top 50 drugs. To have more reliable protein linkages, we considered only interactions whose *combined score* was greater than 500 in a range of values [0, 1000] representing the 9.6% of the full proteins interactions. Starting with the gene targets of the prioritized drugs and with the genes of the consensus signature, we find the intersections between these two lists and the genes in the PPI. The results of this intersection were plotted using Gephi (ver 0.10.1),^3^a software for graph visualization. The codes for the drug prioritization and the construction of the network are available at the repository https://github.com/ceccarellilab/MEDIAproject.

### Selection of the consensus genes for validation in vitro experiments

Starting from the 39 up-regulated genes of the consensus signature, we selected the 10 genes closest to the drug targets over the network (directly connected), and those still unknown in the literature for experimental validation on human articular chondrocytes.

#### Primary cell cultures

Human Cryopreserved chondrocytes, Osteoarthritis (#CDD-H-2610-OA, CliniSciences) and Human Cryopreserved chondrocytes, Normal (#CDD-H-2610-N, CliniSciences) were cultured in Chondrocyte Growth Medium with 10% Human Serum supplement (#M2600-10HS, CliniSciences) according to manufacture recommendations. The cells were maintained in an incubator at 37 °C and 5% CO_2_ in a fully humidified atmosphere.

#### RNA extraction and real-time PCR

Total RNA was isolated from primary cell cultures using Trizol Reagent (Invitrogen, Carlsbad, CA, USA) and quantified using NanoPhotometer NP80 (Implen, USA). One $\upmu$g of RNA was reverse-transcribed in cDNA using SuperScript VILO (#11754050, Invitrogen) according to the manufacturer’s protocol. The mRNA levels of the analyzed genes were measured by RT-qPCR amplification using iQ SYBR GREEN Supermix (#1708882, Bio-Rad Laboratories) according to the manufacturer’s instructions. We performed RT-qPCR experiments using C1000 Touch Thermal Cycler (Bio-Rad, Hercules, California, CA, USA). The reaction volume was 25 $\upmu$L. Each reaction was performed in duplicate. We quantified mRNA expression using the comparative $\Delta \Delta$Ct method, and we used the Ribosomal Protein S18 gene (RPS18) as a control to normalize the gene expressions. Two independent experiments were performed. Data were analyzed using Biorad CFX Maestro version 1.0 (Bio-Rad). For the complete list of the oligonucleotides used for the RT-qPCR, see Additional file 1.

### Data pre-processing to perform the patient-centric strategy

The patient-centric approach aims to determine a risk score to distinguish between healthy and OA individuals and capture the disease severity for each patient with a value. We fitted a logistic regression model to reach this goal.

We chose Dataset 3 as the *training* set for fitting the model and Dataset 4 as *test* set to demonstrate the approach’s efficacy. We used the $\log _2(TPM)$ normalized counts since they represent a measurable physical quantity readily available for each patient without the need for between-sample normalization. As a proxy for the TPM values related to the training set, we used the matrix returned by Soul and colleagues from the tximport processing, where the TPMs have been computed using the effective gene lengths [25, 37]; we selected genes with TPM > 0 for at least the 50% of all the samples, dealt for gene isoforms by computing the mean TPM values per-patient, and then applied $\log _2$ transformation. For the validation dataset, we computed the TPMs starting from raw counts and collecting the theoretical gene lengths by the EDASeq R package [53], filtered out null values as performed in the training dataset, and $\log _2$ transformed the data.

We performed the following pre-processing steps on the data prior to the model implementation and score extraction:

1. At first, we considered all the genes in common between the two datasets (the training set and the test set).
2. We applied the quantile normalization transformation to the training set.
3. Finally, we re-scaled each sample in the test set to follow the distribution of the quantile normalized $\log _2(TPM)$ values in the training cohort.

All the details about the procedure used for this strategy and the corresponding R code are available at https://github.com/ceccarellilab/MEDIAproject.

### Models set-up for risk score computation

We defined a reduced signature-based risk score $s_R$ by using logistic regression with Elastic-Net penalization to achieve feature selection and identify a reduced subset of consensus genes retaining the maximum information associated with OA.

To determine whether using a small number of features might compromise the efficacy of the score, we also evaluated the total signature risk score $s_T$, incorporating all of the consensus genes within a logistic regression with the Ridge penalization. Ultimately, we compared the two scores’ classification performances to examine how well they captured the OA condition. The user can reproduce the score computation on this data or other dataset by using the code available at the vignette (see subsection above).

#### Elastic net model to extract highly informative features

We fitted a logistic regression model with an Elastic-net penalty using the caret R package [54] with method =“glmnet” and $\alpha$ = 0.5, to select the genes of the consensus signature that were most relevant in discriminating OA patients from healthy ones. Due to the class disbalance and the small number of samples available for the training, we applied a bootstrapping strategy with Leave-One-Out-Cross-Validation (LOOCV). We trained the algorithm on *n *= 100 sub-datasets, composed of the 10 healthy patients available from the cohort and as many affected patients randomly selected for each sub-dataset. The resulting model returned the vector of estimated regression coefficients for each run with the non-significant coefficients set to zero. As the reduced signature, we selected only the genes detected as significant in at least 50% of the runs. Then, we averaged the coefficients associated with this reduced signature across all the 100 models to use these mean values to compute the final score $s_R$ for each patient *i* as shown in (1).

1 $$\displaystyle {s_{R}}(i)=\sum_{j=1}^{m}a_j*z_{j,i}$$

In this formula, $a_j$ is the average value of *j*-th significant regression coefficients (i.e., *j*-th gene in the reduced signature of length *m*) computed across all the runs, and $z_{j,i}$ is the $\log _2(TPM)$ value of the corresponding gene *j* in patient *i*.

We used the Receiver Operating Characteristic (ROC) curve and related Area Under the Curve (AUC) to assess the discriminative capability of the reduced signature.

To further evaluate the $s_R$ score, we computed the z-score transformed $\log _2(TPM)$ values and performed an unsupervised clustering on the expression of the consensus genes available for both the training and test datasets. Since we added the class and the assigned $s_R$ score annotations, we sorted all the samples by increasing assigned score values in the heatmaps to estimate the concordance with the corresponding class.

#### Ridge regression on all the features for assessing method reliability

We calculated the total risk score, $s_T$, considering all the features of the consensus signature and applied a logistic regression with the Ridge penalization. We used the LOOCV as previously described. Since Ridge penalization did not perform a feature selection, the model estimated the vector of coefficients for all genes in the signature. Using these coefficients and the corresponding expression values, we derived $s_T$ as

2 $$\displaystyle {s_{T}}(i) =\sum_{j=1}^{k}b_j*z_{j,i}$$

where $b_j$ is the estimated regression coefficient for the gene *j* of the consensus signature of length *k*. We evaluated the ROC curves and AUC for the $s_T$ risk score on the training and test sets. Finally, we used the DeLong test to assess if there was a significant difference between the two AUCs [55].

## Results

### Differential analysis reveals common biological programs activated in OA samples across heterogeneous datasets

**Fig. 2:**
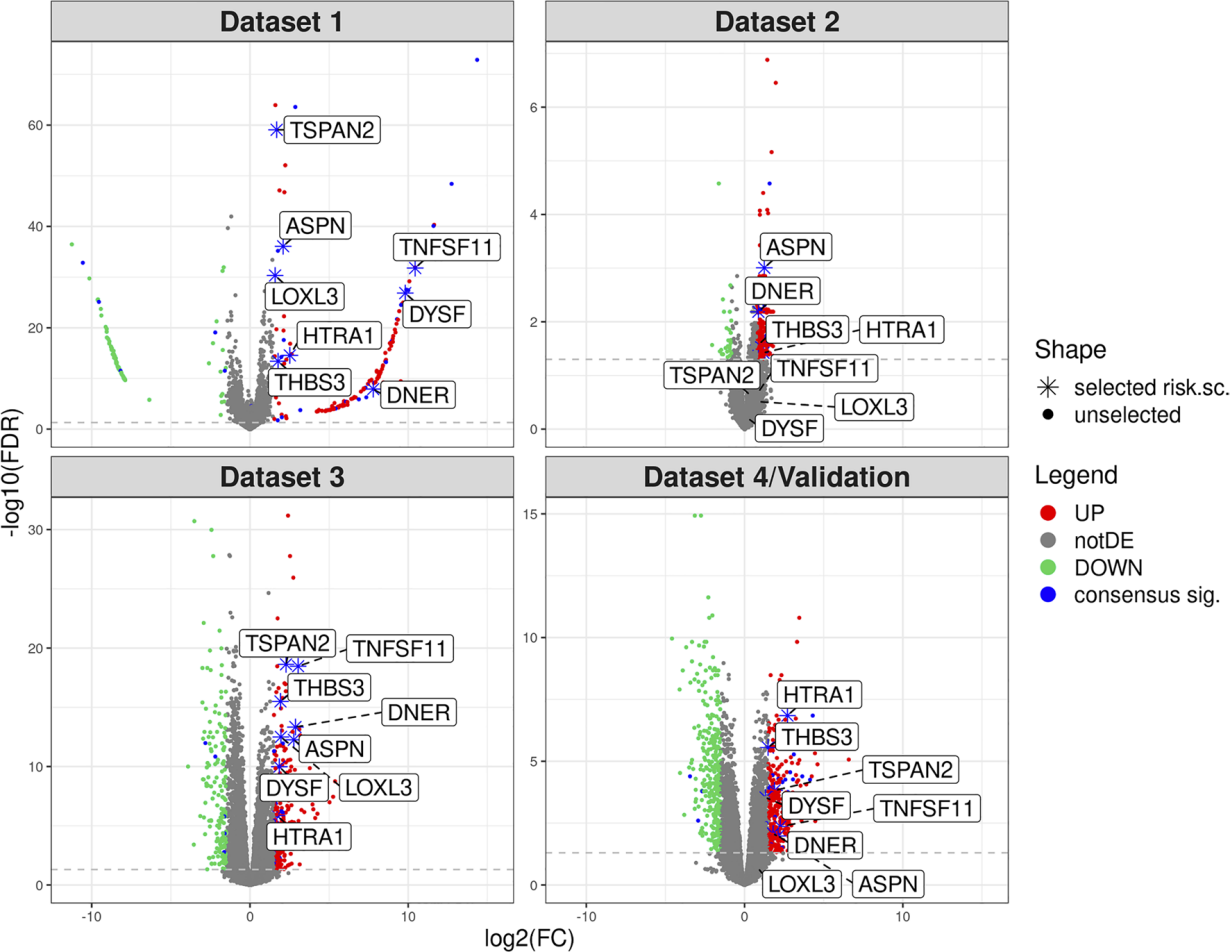
Volcano plots for all the datasets considered in this study. DE genes identified in each cohort are respectively in red and green as up/down-regulated genes; the genes in the consensus signature are in blue; not DE genes in grey; the genes chosen for the risk score procedure are labeled and shaped with an asterisk

Investigating the DE analysis within each cohort, we obtained an overview of the biological processes and pathways characterizing OA patients compared to healthy controls accounting for the diverse experimental setups of each study. For example, we observed a different number of significantly dysregulated genes: 156 up, 79 down for Dataset 1, 190 up and 28 down for Dataset 2, 272 up, 170 down for Dataset 3, 315 up, 320 down for the Validation dataset (see Fig. 2 and Additional file 2).

**Fig. 3:**
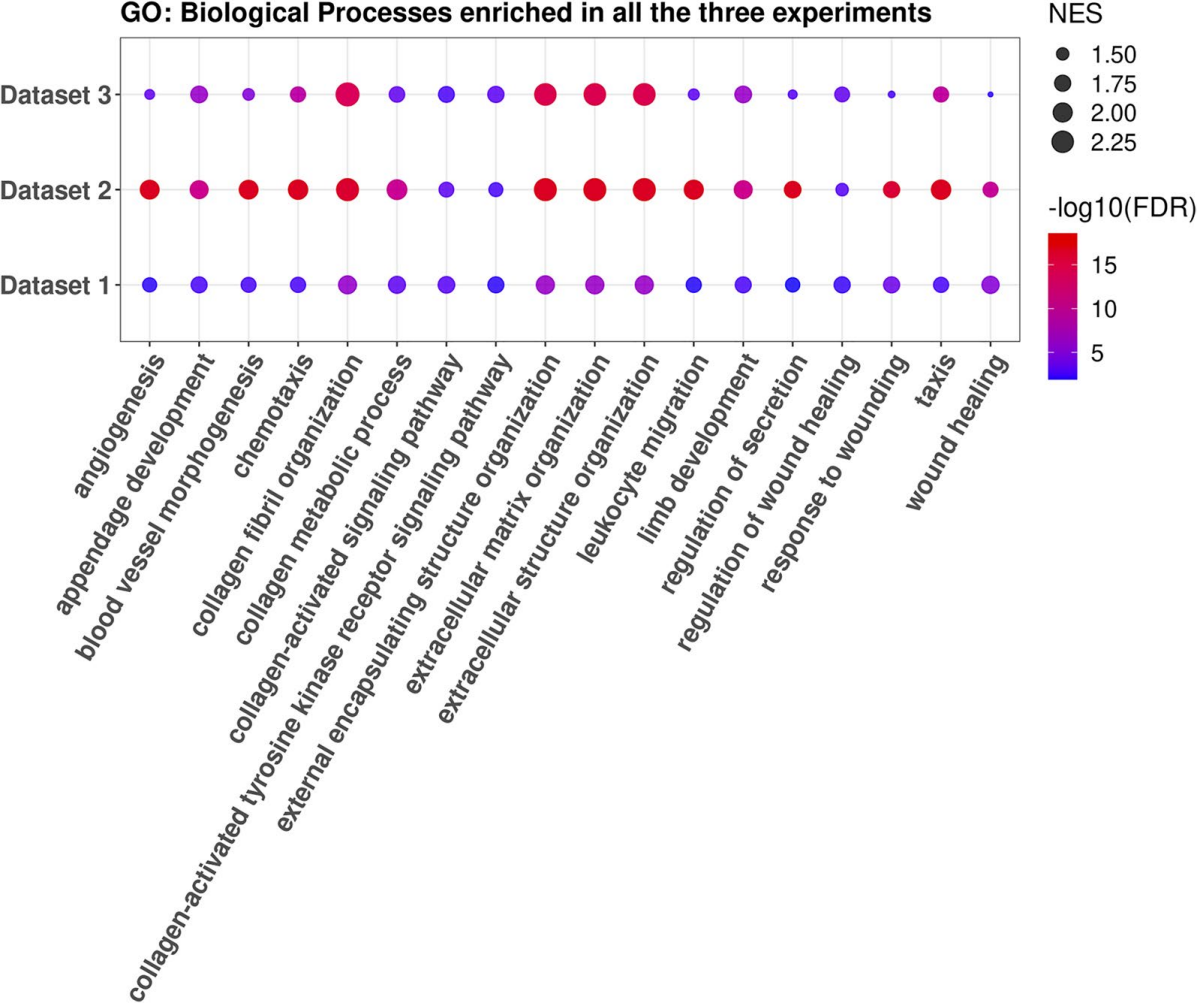
Bubble chart of commonly enriched biological processes. The figure shows the significantly enriched GO:BP terms shared by all the up-regulated genes for the three cohorts. For each cohort, the size of the dots is the normalized enrichment score (NES) and the color is the statistical significance as $-\log _{10}(FDR)$

Nonetheless, the GSEA analysis showed substantial agreement in the altered biological processes among the different cohorts. For example, the GO terms “Extracellular Matrix Organization” and “Extracellular Structure Organization” were found to be significantly enriched in the OA profiles across the three signatures (Fig. 3). The analysis of the REACTOME pathways provided similar insights, including pathways related to collagen fibril synthesis and reorganization as the most enriched (Fig. 3, Additional file 4: Fig. S1).

### A *consensus* signature characterizing the OA phenotype

**Fig. 4:**
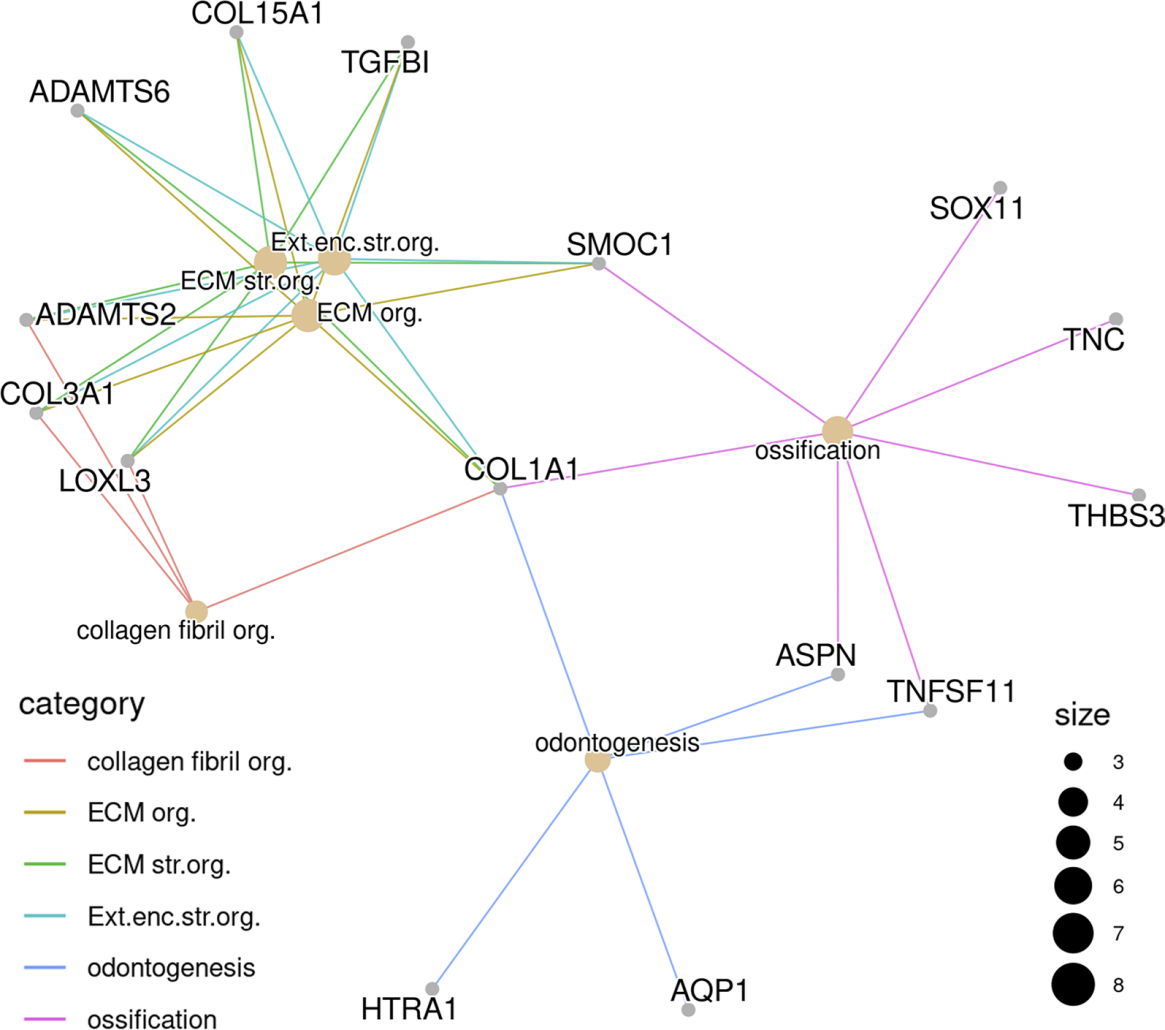
Consensus signature enrichment analysis. The CNET plot shows the top 6 GO:BP enriched by the up-regulated genes of the consensus signature (over-representation analysis with hypergeometric test); the category node size is the $-log_{10}(FDR)$ returned from the analysis. ECM org.: extracellular matrix organization; ECM str.org.: extracellular structure organization; Ext.enc.str.org.: external encapsulating structure organization; collagen fibril org.: collagen fibril organization

**Fig. 5:**
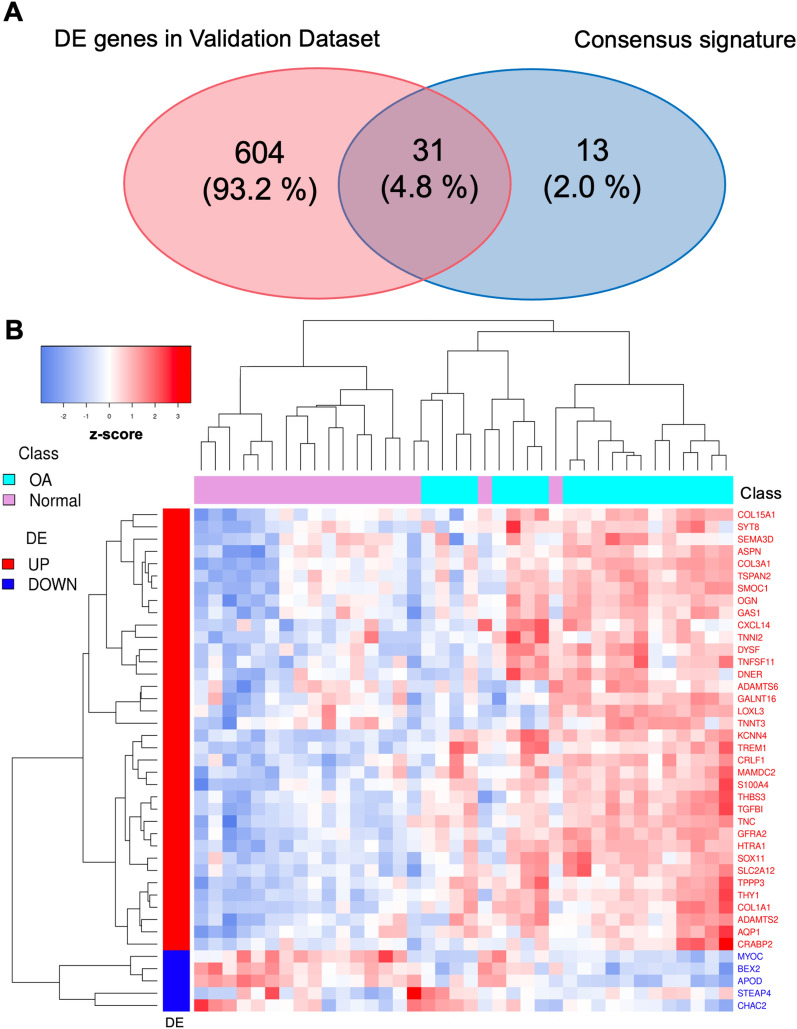
Consensus signature validation. **A** Venn plot representing the overlap between DE genes in the validation dataset and the consensus signature (P $<<$ 0.01); **B** the heatmap showing the expression of the consensus genes (DE) across the samples of the validation cohort (z-score)

The meta-analysis of the three cohorts allowed us to identify a consensus signature including 44 genes (39 up, 5 down) that better recapitulate the coherent differences associated with the OA (Additional file 3: Table S1). Enrichment analysis of the consensus profile yielded extracellular matrix and bone reorganization-related terms as the most enriched by the up-regulated genes (Fig. 4). We assessed the consensus signature, evaluating its agreement with an independent cohort [26]. The Venn diagram in Fig. 5A, shows that 31 out of 44 consensus signature genes were also DE in the validation dataset. The hypergeometric test revealed that the overlap was statistically significant (p-value $< 0.01$). As further validation, the unsupervised clustering applied to the validation dataset and induced by the consensus genes confirmed their discriminative power (Fig. 5B, 3 out of 44 genes were excluded after the pre-processing). Also, the GSEA Running plot shows that consensus signature genes tend to appear toward the top of the validation dataset expression profile (Additional file 5: Fig. S2).

### Drug prioritization and network analysis uncover potential pharmaceutical targets

**Fig. 6:**
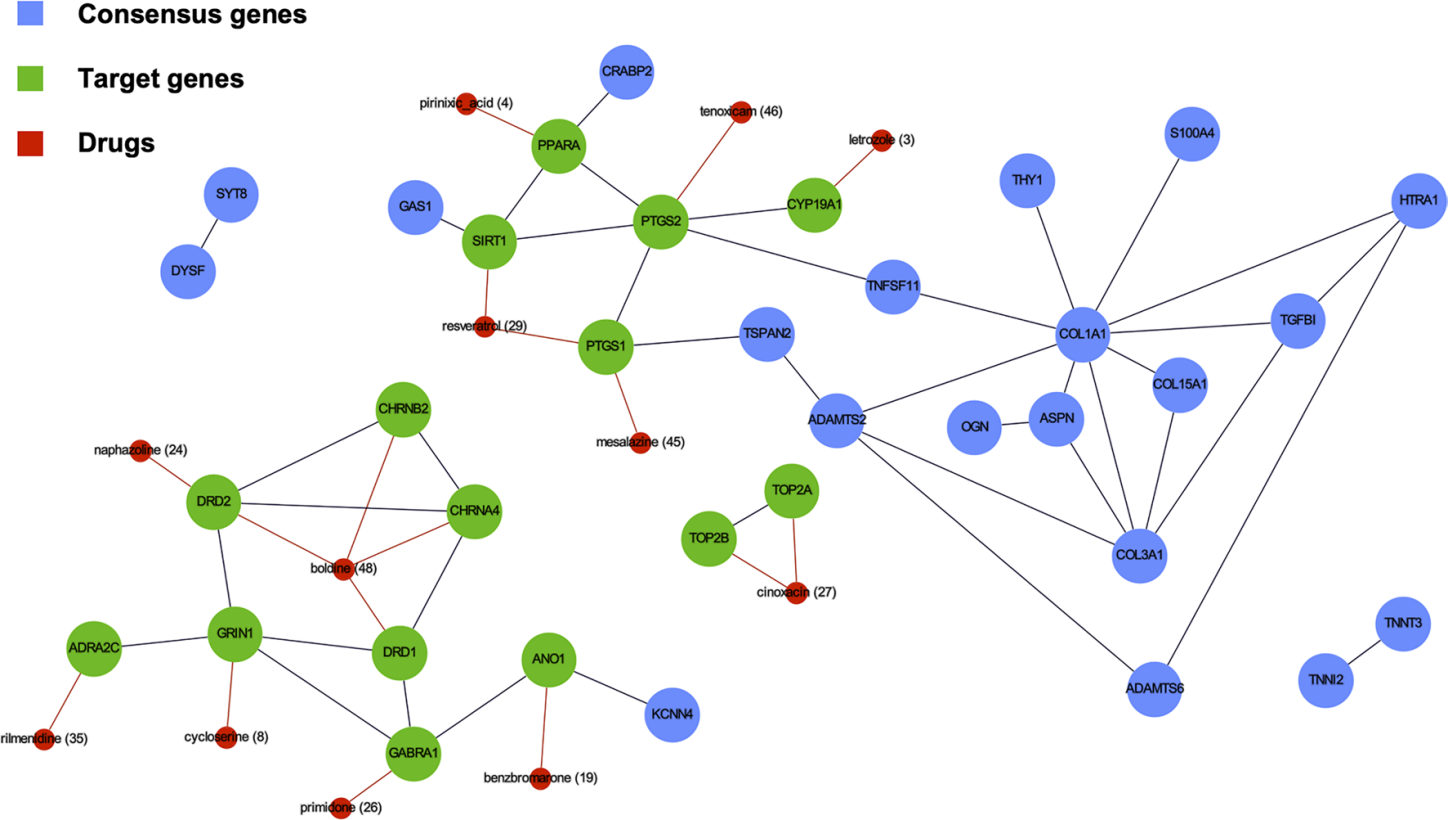
Network construction. The figure shows a sub-graph extracted from the PPI graph filtered by considering only DE up consensus genes (blue) and the targeted genes (green) of the top 50 drugs (red, rank value reported). Those links represent the STRING *combined score* greater than 500. Single genes that had only low-scoring connections or none of them, and our Genes that did not match those of STRING, were not shown

In the *disease-centric* analysis, we scored a list of ~ 19,000 drug-induced gene expression profiles based on their predicted ability to counteract the transcriptional effects induced by OA. In particular, the top hits in this approach correspond to those drugs that can up-regulate the pathways involving genes that are down-regulated in the OA signature and vice-versa. This prioritization is entirely target-agnostic but still carries transcription-related information, which we sought to exploit in identifying potential clinically relevant genes. We also performed a PPI network analysis, including the signature genes and top drug molecular targets. In particular, we first noted that none of the molecules in The Library of Integrated Network-based Cellular Signatures (LINCS) database^4^ had any of the OA signature genes as their targets. Then, we selected relevant genes from the consensus signature based on the assumption that those appearing along a short path to a drug target could have clinical significance (Fig. 6). Moreover, 5 genes (*PPARA, SIRT1, PTGS1/*2*, ANO1*) that are direct targets of several prioritized drugs showed a direct interaction with signature genes. Among the corresponding drugs, Resveratrol has been shown to exert positive effects on tissues’ homeostasis both in vitro and in vivo [56–58]; Tenoxicam is currently used as a treatment for pain and inflammation relief in various degenerative rheumatic diseases [59, 60]. Other relevant drugs revealed by the network analysis were Benzbromarone, Pirinixic Acid, and Mesalazine (see “Discussion” section).

### Experimental validations

The reduced set of genes we extracted for the in vitro validation included *TSPAN*2, *TNFSF11, GAS1, KCNN*4 and *CRABP*2 as the closest in the network, and *THY1, TGFBI, S100A*4 *HTRA1* as additional genes. Furthermore, we included *COL*3*A1* as a positive control to assess the test validity of the experimental protocol and to confirm literature results on Primary Human Chondrocytes.

**Fig. 7:**
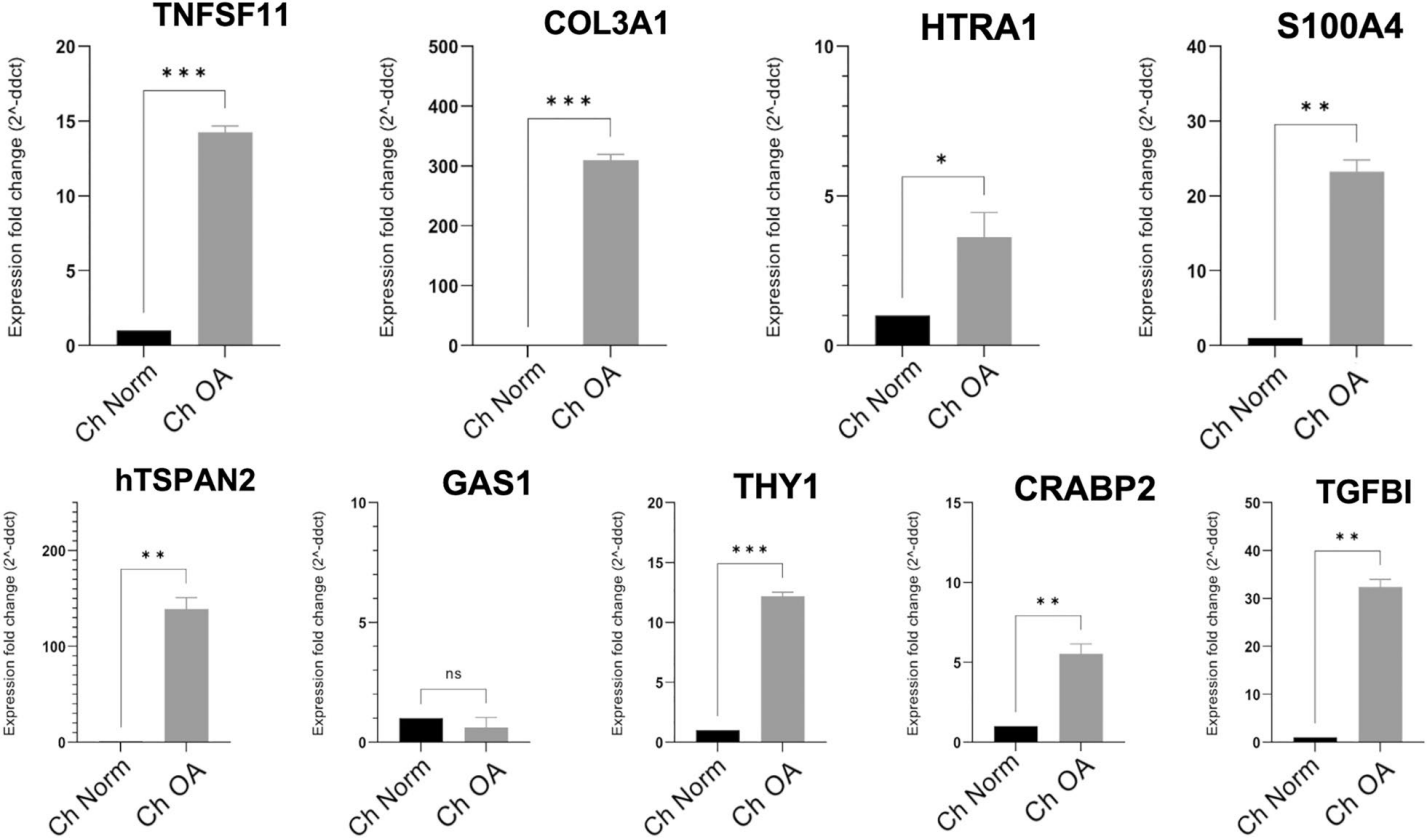
In vitro validation. mRNA fold expression was evaluated in normal and OA chondrocytes for 9 of the 10 selected consensus genes: *TNFSF11*, *COL*3*A1*, *HTRA1*, *S100A*4, *TSPAN*2, *GAS1*, *THY1*, *CRABP*2, *TGFBI*, using unpaired t-test (*P < 0.05, **P < 0.01, ***P < 0.001)

For details on the gene selection, see Additional file 1. Results from RT-qPCR experiments agreed with in silico predictions. Specifically, Fig. 7 shows that most of the tested genes had marked overexpression in OA cells compared to normal cells, except for *GAS1* and *KCNN*4 (not shown because of undetected signal). Among the validated genes, *TSPAN*2, *HTRA1* and *TNFSF11* are also part of the sub-signature extracted for the risk-score computation (see next paragraph).

### A single patient risk score to predict OA status

**Fig. 8:**
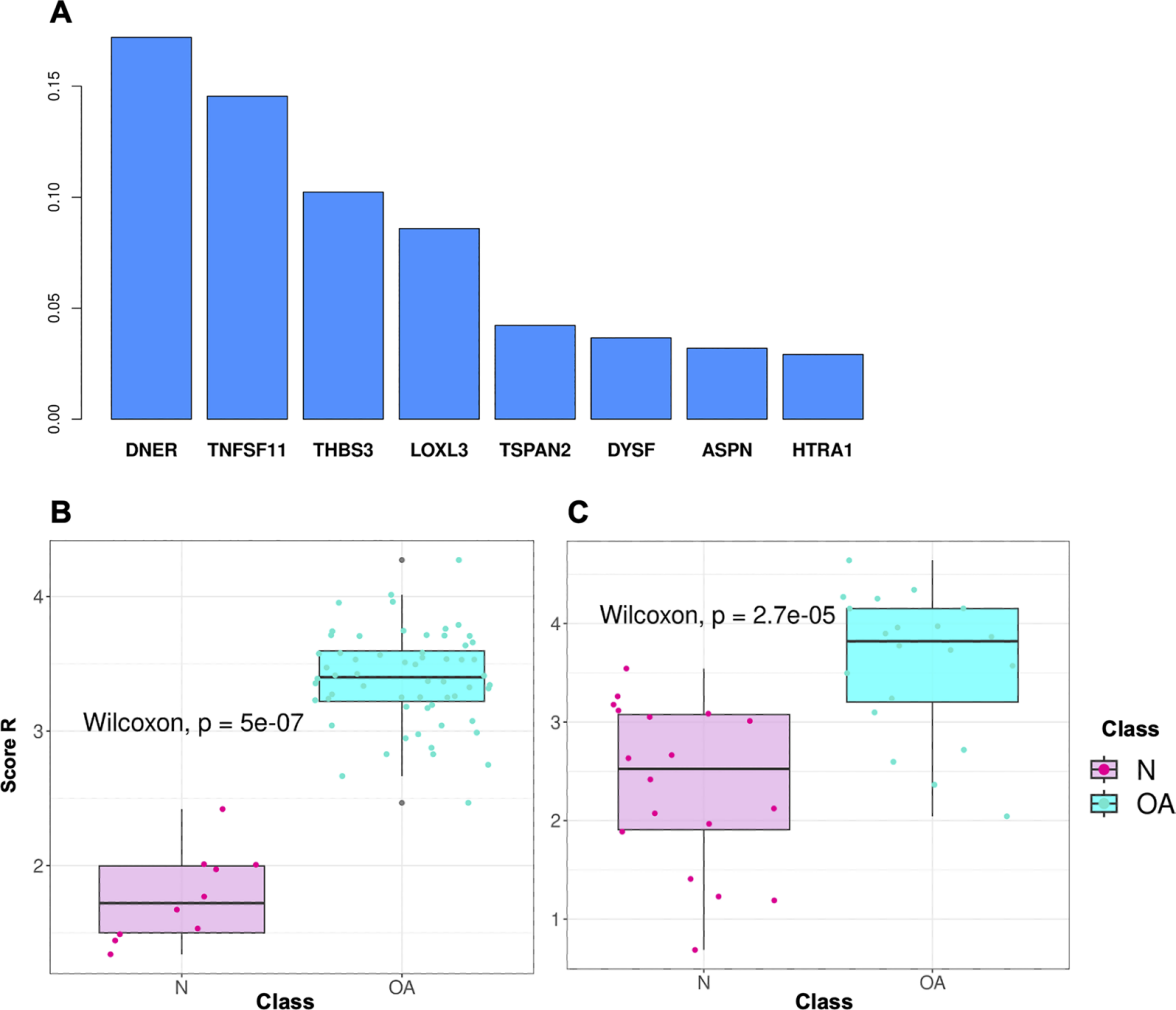
Risk score computation based on the most discriminating genes of the consensus signature. In **A** the barplot shows final mean coefficients associated with their related genes, sorted by decreasing value; in **B** and **C**, boxplots report the $s_R$ score distributions, associated with the training and test sets respectively

**Fig. 9:**
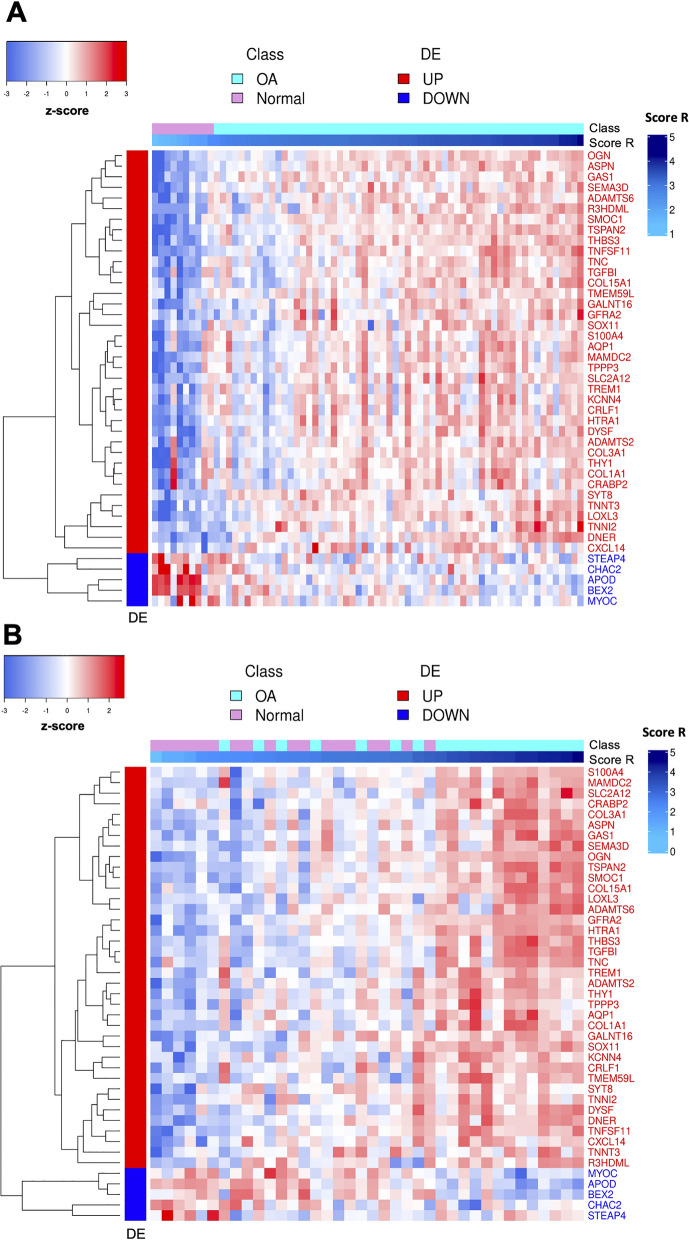
Validating risk score. In **A** and **B**, the heatmaps of the consensus genes expression (DE), where the patients are sorted by increasing risk score, respectively for training and test sets (z-score)

**Fig. 10:**
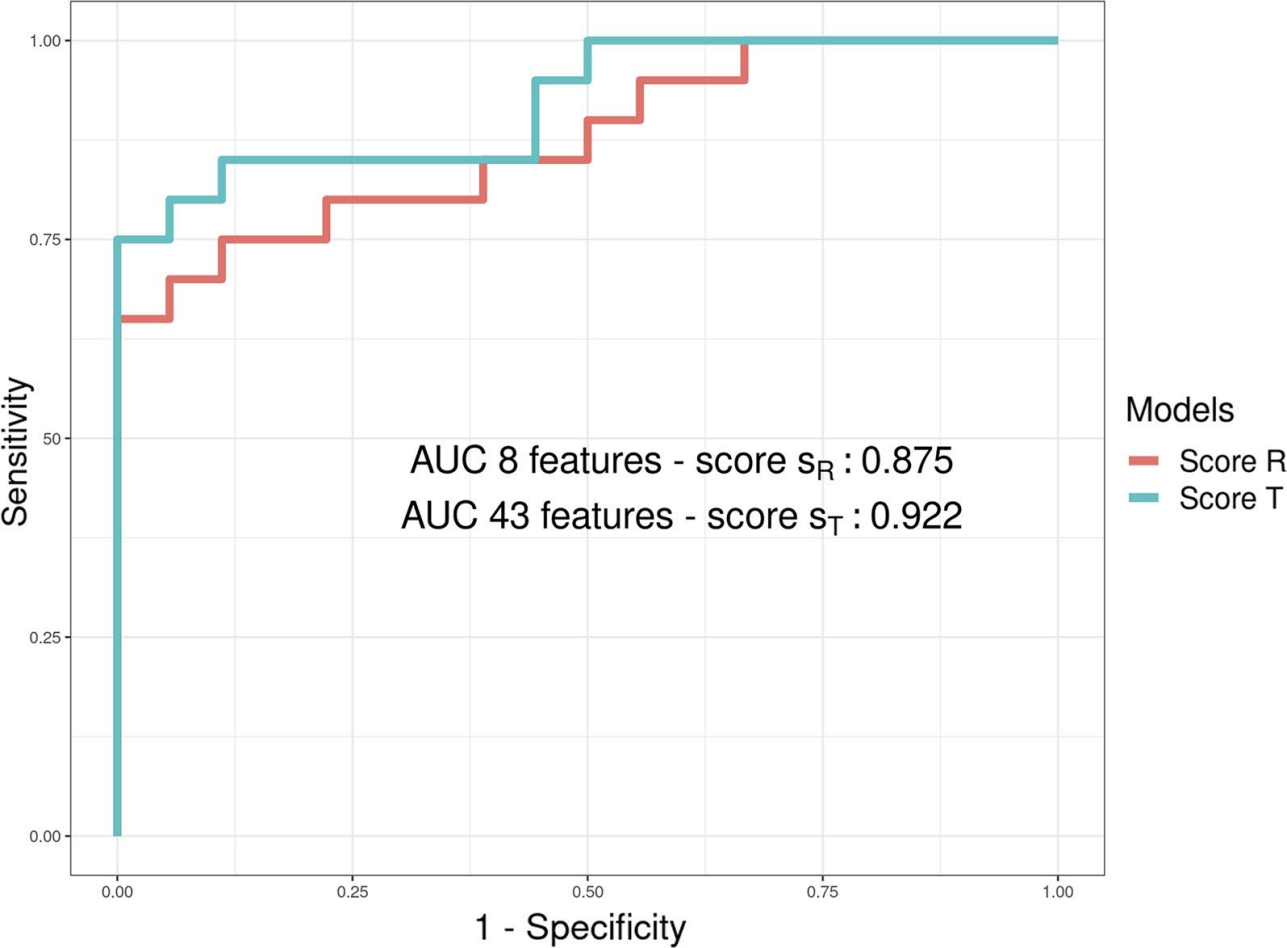
Performance comparison (Validation dataset) of the two models. In this plot we see that the AUC for the $s_R$ score (orange) is not particularly lower than the AUC returned from the $s_T$ score (turquoise); we confirm that reducing the number of features used for the score calculation does not affect the risk estimation of the score

The *patient-centric* analysis allowed us to derive risk scores associated with OA. For what concerns reduced $s_R$ score, the logistic regression with the Elastic Net penalty and the bootstrapping strategy allowed us to identify *DNER, TNFSF11, THBS*3*, LOXL*3*, TSPAN*2, *DYSF, ASPN* and *HTRA1* as the features selected in at least the 50% of the runs (Additional file 6: Fig. S3). Such genes are up-regulated as consensus genes (Fig. 2). The weights associated with such genes are used to evaluate $s_R$ (Fig. 8A). After evaluating $s_{R}$ for each sample *i* in both the training and the test data, we compared the score distribution across the two classes of patients: Fig. 8B and C shows that Normal and OA samples have a significantly different $s_R$ score distribution in both training and test cohorts (p-values $<<$ 0.01, Wilcoxon test). Moreover, Fig. 9A, B shows that patients sorted by their $s_R$ scores tend to segregate both for disease status and expression of the signature genes. While the segregation is perfect in the training set (panel A), it is also highly evident in the test set (panel B). Finally, we compared the classification performances of the $s_R$ score against that of an analogous score (referred to as $s_T$) based on the consensus signature genes available in both datasets (43 out of 44). The corresponding ROC curves reported in Fig. 10 show that using a reduced set of genes does not significantly impact the prediction performance (AUC equal to 0.875 as compared to 0.922, the DeLong AUC test applied returned a p-value of 0.507).

## Discussion

The OA patient phenotype is very varied, although it shares a progressive and unrestrainable degeneration. While significant progress in treating clinical manifestations has been made, fundamental molecular mechanisms underlying the disease remain poorly understood, thus limiting the possibility of identifying effective drugs for the restoration of tissue homeostasis. Such a targeted approach would be especially practical if applied as early as possible, minimizing the damage caused by the degenerative and progressive nature of the disease. Our work aimed to contribute to this endeavor by extracting a reduced set of relevant genes from multiple heterogeneous OA-related datasets. Indeed, as expected, each cohort showed its transcriptional specificities. Such variability could be exacerbated by the degenerative nature of the disease, which progressively affects the extracellular matrix and collagen fibrils, depending on the anatomical structures involved. Nonetheless, the integration process allowed us to isolate 44 genes that appear significantly dysregulated across diverse samples. The panel includes previously known OA-related genes such as *COL*3*A1, ASPN* or *ADAMTS6/S*2, that are involved in cartilage remodeling and inflammation with a specific role in the pathology [61–64] or *TSPAN*2 that correlates with inflammation and immune-cell infiltration in affected cartilage [65] and is overexpressed in OA cartilage of murine models [66].

Intriguing is the putative role of the retinoic acid binding protein *CRABP*2 as a target: the expression of this gene was demonstrated to be sensibly higher in murine models with degenerative joint disorders [67]. Furthermore, fibroblast-like synoviocytes (FLS) derived from rheumatoid arthritis patients showed augmented apoptosis in response to gene inhibition [68]. *TNFSF11* has been recently associated with the induction of the PI3K/AKT/mTOR signaling in affected tissues, supporting inflammation and structure destruction: in particular, its downregulation could enhance mitophagy and reduce cartilage degradation [69]. The crucial role that this gene holds in bone remodeling makes it a potential therapeutic target in the treatment of OA and other degenerative joint diseases [70, 71].

Although not known to be directly linked to the disease, other genes could be worthy of further investigation. For example, *DNER* has been primarily detected in OA-affected cartilage, and its role in the disease is currently under investigation [72, 73]. Indeed, we extracted novel genes such as *S100A*4, which is known to be involved in cancer metastatic progression but that results overexpressed in cartilage and synovium damaged by arthritis [74, 75]. *HTRA1*, which has a role in various biological processes, including cancer, is intriguing for its involvement in musculoskeletal diseases [76, 77].

Even if most of the genes in our panel are not known to be direct targets of any drug, our network-based analysis allowed us to identify the most proximal ones, which may provide clues into the therapeutic modes of action for the prioritized drugs. Among the genes that are close to the drug targets, *PTGS1/*2 are pivotal in the inflammatory process induction (involved in prostaglandin synthesis) and may have clear implications in the pathology progress [78, 79]. On the other hand, *SIRT1* and *PPARA* have a protective and anti-inflammatory action when targeted by their activators and agonists [80–83]. Indeed, PPARA is a PI3K/AKT/mTOR signaling regulator, promoting autophagy by mTOR signaling pathway inhibition [80, 84, 85]. It was demonstrated that a hypoxic microenvironment can enhance mitophagy and ameliorate chondrocyte viability [85, 86]. Of interest is the role of *HIF-1*$\alpha$ in maintaining hypoxia and slowing cell degeneration in mice OA models [87]. To date, non-steroidal anti-inflammatory drugs, glucocorticoids, opioids, chondroprotective agents and anti-cytokines are the classes of drugs currently used to treat OA accompanied by several side effects for the patient [88, 89]. Some of these, like the most common Prednisolone [90] or Paracetamol and Aspirin [91], induce anti-inflammatory and immunosuppressive effects that block the production of pro-inflammatory cytokines, leukocyte recruitment, and activation. Resveratrol, ranked 29 in our prioritization, could play a role in both prevention and treatment of OA, due to its anti-inflammatory, chondrogenic matrix-protective, or antiaging effect [56–58, 92–94]. Even an anti-gastric cancer effect of this molecule has been recently assessed [95]. Mesalazine (ranked 45) and Pirinixic Acid, a potent *PPARA* agonist, (ranked 4) have been shown to have anti-inflammatory potential in OA and may warrant further testing [96, 97]. Finally, Benzbromarone (ranked 19) is a medication used to treat gout [98, 99], but we do not have evidence for using this drug for OA treatment. The drug prioritization analysis helped identify a set of genes that may be clinically relevant as both part of the consensus signature and close to the top drugs in the PPI network. The RT-qPCR results confirmed a significant dysregulation of seven of the ten genes we selected for validation.

Although a reduced panel of 44 genes may represent a valuable reference for future mechanistic studies, a smaller number is required to achieve practical relevance in clinical settings. For this reason, in the *patient–centric* approach, we aimed at developing a score to assess the disease severity with a limited number of genes. The $s_R$ score, based on only 8 genes, was proved effective and with statistically similar accuracy to $s_T$ obtained with the larger panel of 43 genes. Together with additional clinical parameters, this score could be helpful in rapid preliminary checks and assessment of disease progression. This study has a few limitations. The reliability of the results could be affected by the limited number of samples available for the training phase of both models. However, we tried to assess this risk using bootstrapping steps and by including a test dataset. Moreover, the specificity of the selected samples, all from patients with damaged cartilage, may limit the possibility of generalizing the result to all OA phenotypes. While we used prioritized drugs as proxies to identify clinically relevant genes, their actual effectiveness as OA treatments would require additional efforts that fall out of the scope of this study. Nonetheless, our results show that some of them, such as Resveratrol, warrant further investigation.

## Conclusions

Using a meta-analysis of several OA cohorts, we first identified a panel of 44 genes (39 up-regulated and 5 down-regulated) consistently dysregulated across the different studies, therefore carrying robust molecular features that could be critical for understanding the disease’s mechanisms and suggesting novel therapies. Therefore, starting from this panel and using a network-based approach, from one side, we identified a few drugs that could enlighten therapeutical approaches for OA. For such purpose, we prioritized drugs that can revert the profile and whose targets are close to the identified genes. With this approach, we found either drugs already tested in OA or potential novel therapeutic approaches. Conversely, we extracted a subset of eight genes and defined a patient-specific risk score with significant prediction power to distinguish OA from healthy individuals. The presented results could help understand OA mechanisms from multiple perspectives, including its pathophysiology, diagnosis, and treatment. However, we employed four cohorts related to a specific subtype, cartilage, with a limited number of patients due to the scarcity of publicly available data specific to OA (compared to other studies). It would be interesting to validate our findings’ generalizability more in-depth (for example, when varying clinical and demographic data) and enhance the signature and score obtained with additional data. An example could be the application of this framework on other tissue types like synovial fluid or subchondral bone [23, 100]. The proposed methodological approach can easily scale to additional cohorts of assessment. Therefore, future dataset availability could support the translational purpose of our work.

### Supplementary Information

**Additional file 1.** Oligonucleotides designed as primers and information related to the genes selected from the network to set up the validation experiment.

**Additional file 2.** DE genes derived from each DEA related to the three datasets.

**Additional file 3.** The consensus signature of 44 genes.

**Additional file 4: Figure S1.** Enrichment Pathway analysis from REACTOME. Significant pathways enriched by DE genes for each experiment.

**Additional file 5: Figure S2.** GSEA running plot. The positions of the 44 genes in the ranked list of DE genes of the validation dataset are set on the abscissa axis, while on the ordinates is the calculated enrichment score. The consensus signature is significantly enriched (P << 0.01).

**Additional file 6: Figure S3.** Feature selection by means Elastic net bootstrapping. In this plot, for each feature on the ordinate axis, we marked (red) each run in which it was selected by the model. We finally chose the features occurring in at least 50 runs.

## Data Availability

All the analyses performed have been done using RStudio with R version 4.2.1 (2022–06–23) and Python version 3.9.7. The code of the implemented pipeline and supplementary files useful to reproduce the analyses can be found at: https://github.com/ceccarellilab/MEDIAproject. The vignette is related to the entire pre-processing workflow of the datasets, consensus signature extraction, enrichment analyses, and patient-centric strategy. We provided the files with the codes for the disease-centric approach in the repository, also including all the useful information related to the package versions used.

## Footnotes

1 https://genome.ucsc.edu.

2 https://github.com/soulj/OAStratification.

3 https://gephi.org/.

4 https://lincsproject.org/.
