# Supplementary material for: Identification of therapeutic targets in osteoarthritis by combining heterogeneous transcriptional datasets, drug-induced expression profiles, and known drug-target interactions
Source: J Transl Med. 2024 Mar 15;22:281. doi: 10.1186/s12967-024-05006-z (PMC10941480; doi:10.1186/s12967-024-05006-z)
Supplement: Supplementary file 4 — Additional file 4: Figure S1. Enrichment Pathway analysis from REACTOME. Significant pathways enriched by DE genes for each experiment. [file 12967_2024_5006_MOESM4_ESM.pdf]

Supplementary Figure 1

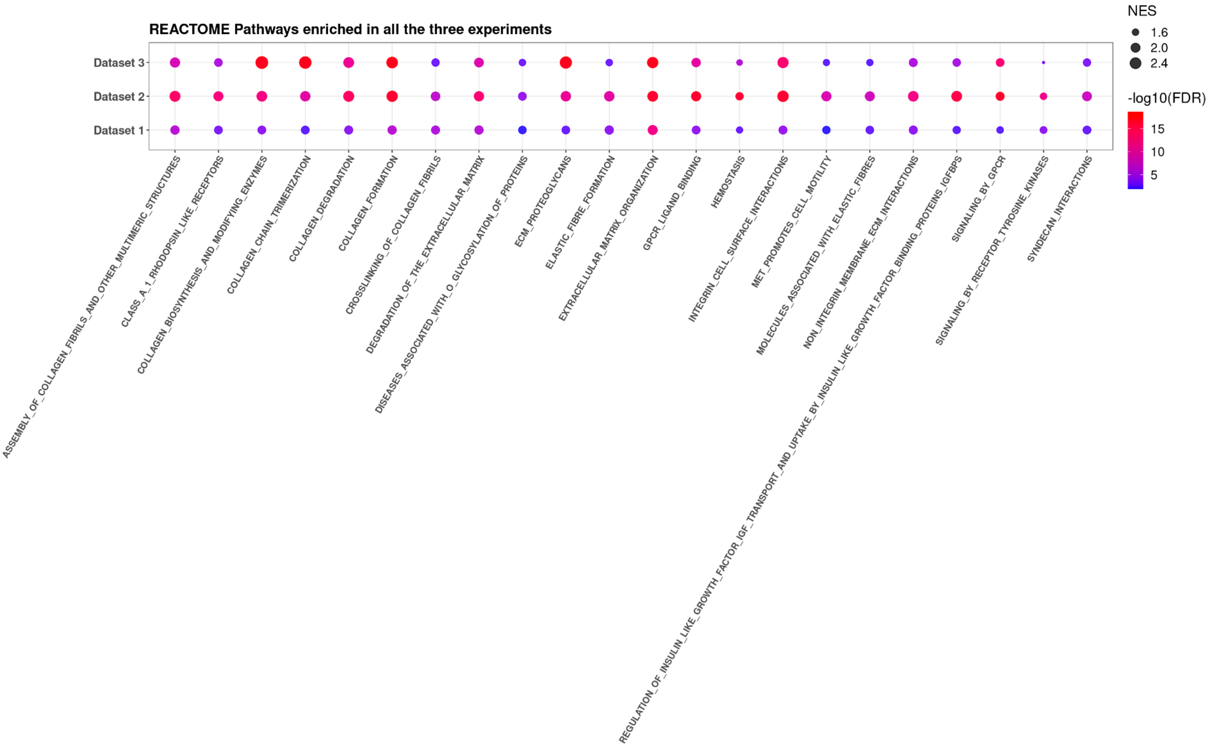

Enrichment Pathway analysis from REACTOME.

Significant pathways enriched by DE genes for each experiment.
