# Supplementary material for: Identification of therapeutic targets in osteoarthritis by combining heterogeneous transcriptional datasets, drug-induced expression profiles, and known drug-target interactions
Source: J Transl Med. 2024 Mar 15;22:281. doi: 10.1186/s12967-024-05006-z (PMC10941480; doi:10.1186/s12967-024-05006-z)
Supplement: Supplementary file 5 — Additional file 5: Figure S2. GSEA running plot. The positions of the 44 genes in the ranked list of DE genes of the validation dataset are set on the abscissa axis, while on the ordinates is the calculated enrichment score. The consensus signature is significantly enriched (P << 0.01). [file 12967_2024_5006_MOESM5_ESM.pdf]

Supplementary Figure 2

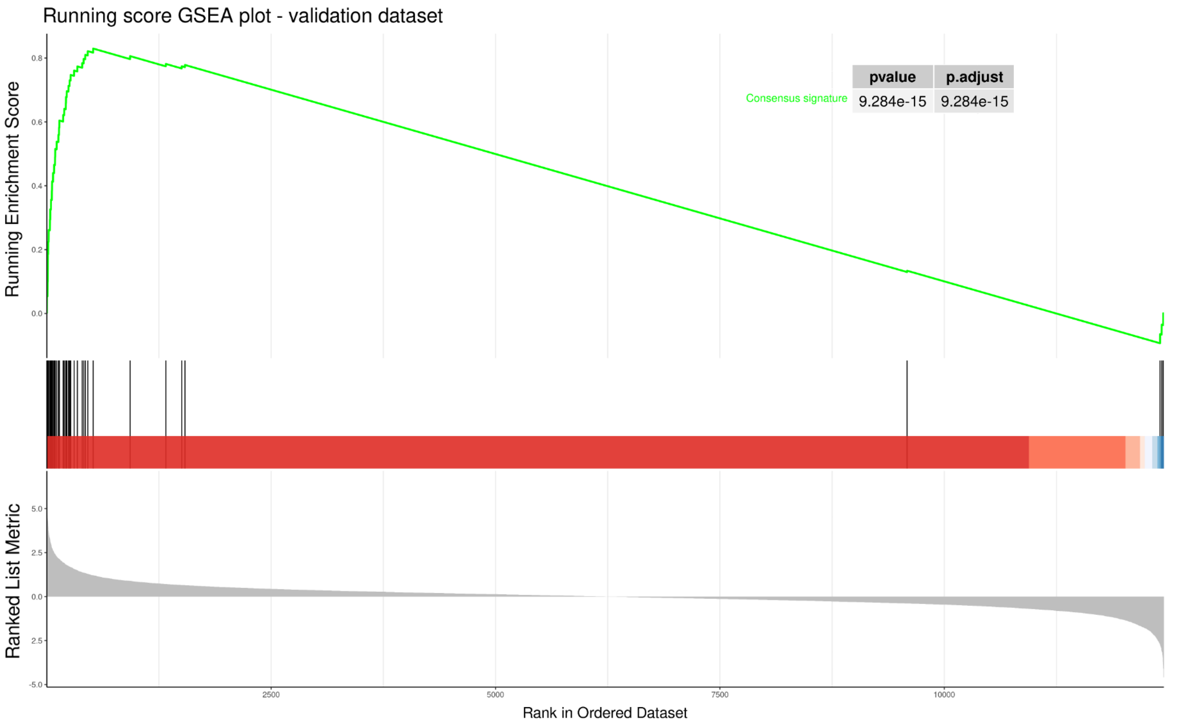

GSEA running plot. The positions of the 44 genes in the ranked list of DE genes of the validation dataset are set on the abscissa axis, while on the ordinates is the calculated enrichment score. The consensus signature is significantly enriched (P << 0.01).
