# Supplementary material for: Identification of therapeutic targets in osteoarthritis by combining heterogeneous transcriptional datasets, drug-induced expression profiles, and known drug-target interactions
Source: J Transl Med. 2024 Mar 15;22:281. doi: 10.1186/s12967-024-05006-z (PMC10941480; doi:10.1186/s12967-024-05006-z)
Supplement: Supplementary file 6 — Additional file 6: Figure S3. Feature selection by means Elastic net bootstrapping. In this plot, for each feature on the ordinate axis, we marked (red) each run in which it was selected by the model. We finally chose the features occurring in at least 50 runs. [file 12967_2024_5006_MOESM6_ESM.pdf]

Supplementary Figure 3

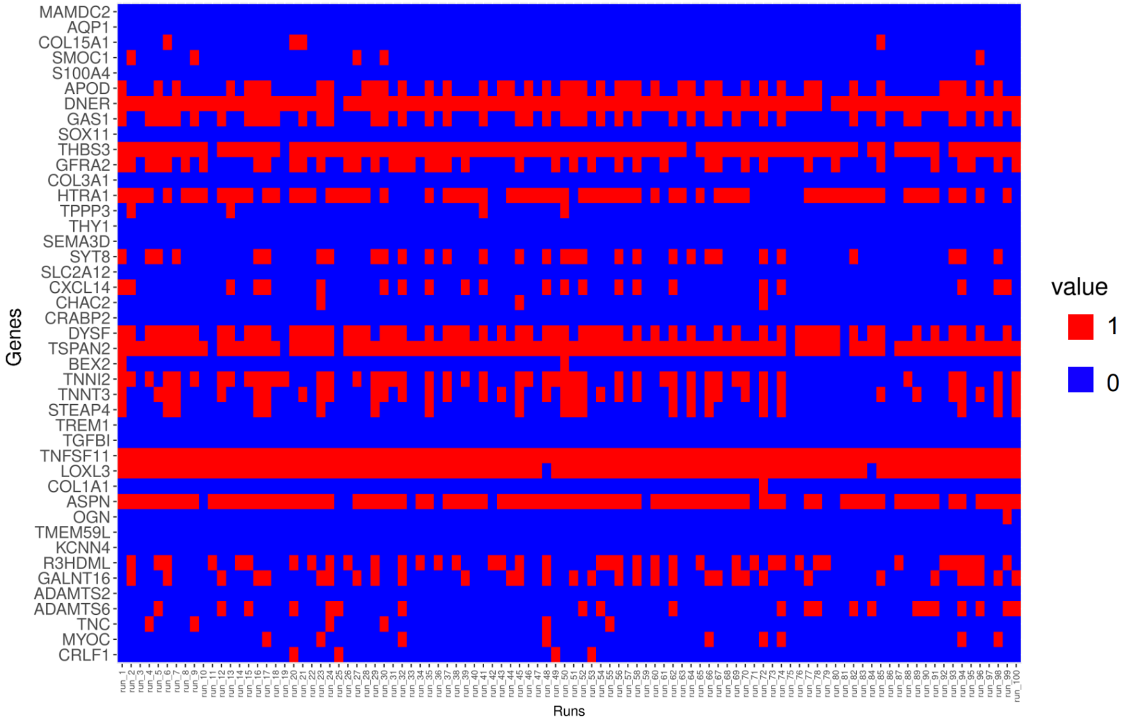

Feature selection by means Elastic net bootstrapping. In this plot, for each feature on the ordinate axis, we marked (red) each run in which it was selected by the model. We finally chose the features occurring in at least 50 runs.
